# Anatomic and Histologic Examination of a Grossly Enlarged Gallbladder Fused to the Inferior Surface of the Liver

**DOI:** 10.7759/cureus.69217

**Published:** 2024-09-11

**Authors:** Aidan J Maxwell, Eric M Lassiter, Benjamin R Klein, Isaac A Arefi, Adel Maklad, Dalia Y Ibrahim

**Affiliations:** 1 Medical Education, University of Toledo College of Medicine and Life Sciences, Toledo, USA; 2 Anatomy, University of Toledo College of Medicine and Life Sciences, Toledo, USA; 3 Neurosciences, University of Toledo College of Medicine and Life Sciences, Toledo, USA; 4 Pathology, University of Toledo College of Medicine and Life Sciences, Toledo, USA

**Keywords:** anatomical variation, biliary tract, gallbladder, histology, liver

## Abstract

The liver is an important organ located on the right side of the abdomen, predominantly responsible for controlling mechanisms related to metabolism, immunity, digestion, detoxification, and coagulation. The gallbladder is an organ present in the gallbladder fossa on the inferior surface of the liver. The main function of the gallbladder is to store and concentrate bile, aiding in fat digestion. In this article, we present a case report of an anomalous gallbladder discovered during cadaveric dissection. The gallbladder appeared to be fused to the inferior surface of the liver and was grossly enlarged. Furthermore, four diaphragmatic grooves were present on the superior surface of the liver. Histologic investigation revealed an acutely inflamed gallbladder wall at the point of fusion, and luminal contents composed mainly of fibrin and degraded blood products. This anatomy could potentially have clinical implications, such as in symptoms of acute and chronic inflammation and right upper quadrant pain. Furthermore, this article adds to the literature a representation of the gallbladder and would be useful in medical education.

## Introduction

The liver and gallbladder are two organs located in the right upper quadrant of the abdomen with major physiologic functions and pathological implications. The liver is implicated in metabolism, immunity, detoxification, synthesis of clotting factors, and digestion of lipids, among others [1]. The gallbladder is an accessory organ that sits in the gallbladder fossa on the inferior aspect of the liver and contributes to the extrahepatic biliary system, primarily functioning to store and concentrate bile [2]. Upon digestion of food, the hormone cholecystokinin is released, resulting in gallbladder contraction and the subsequent release of bile through the cystic duct, into the common bile duct, and finally into the duodenum where it aids in the digestion of fats. The gallbladder is connected to the liver through a structure called the biliary tract, which consists of bile ducts inside and outside of the liver that are involved in the production and transportation of bile [3].

Gallbladder disease is relatively common, estimated to affect 8% and 16% of American men and women, respectively [4]. While females are affected at two times the rate of males, males more often present with complicated gallbladder disease, manifesting as acute cholecystitis, cholangitis, and pancreatitis [5,6]. Complicated gallbladder disease is typically treated with elective cholecystectomy, with postoperative morbidity, mortality, and hospital stay reduced by the laparoscopic approach compared to an open approach [7]. Although gallbladder disease is fairly common, there have been few recorded cases of a massively enlarged gallbladder, with no reports in the literature about a fused gallbladder [8]. This lack of representation in the literature warrants further exploration regarding the causes of this rare anatomical anomaly.

## Case presentation

The current anomaly was identified in a 93-year-old male who died of cardiovascular disease. All other details of past medical history were not available due to the anonymity of the donor program. While conducting a routine educational abdominal cavity dissection at the University of Toledo College of Medicine and Life Sciences, the liver was identified and found to be adhered to the gallbladder. Upon identification of the abnormal anatomy, the liver and gallbladder were removed from the cadaver for further investigation by transecting the portal triad and inferior vena cava. The gallbladder was in a normal anatomical position relative to the liver; however, the superior surface of the gallbladder was fused with the inferior surface of the liver and there appeared to be a congenital absence of the cystic duct. The gallbladder was grossly enlarged and extremely firm, appearing to be filled with some sort of solid material. The sample was sent to histology for further investigation.

**Figure 1:**
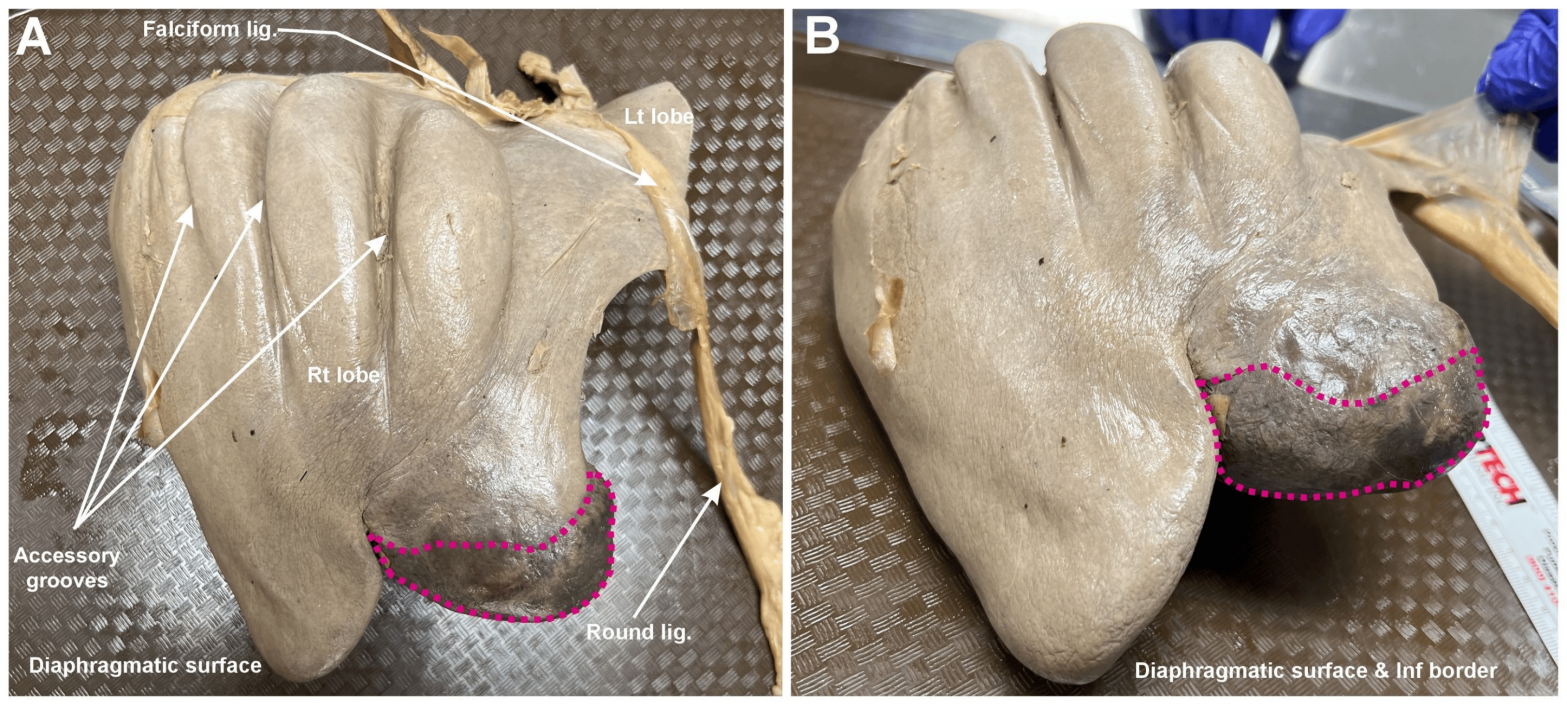
Gross anatomy of the liver and gallbladder. (A) Anterolateral view of the gross anatomy of the fused liver and gallbladder. (B) A lateral view of the diaphragmatic surface of the liver, with the falciform and round ligaments highlighted. The abnormal gallbladder is outlined in magenta. Rt: right; Lt: left; Lig: ligament; Inf: inferior

**Figure 2:**
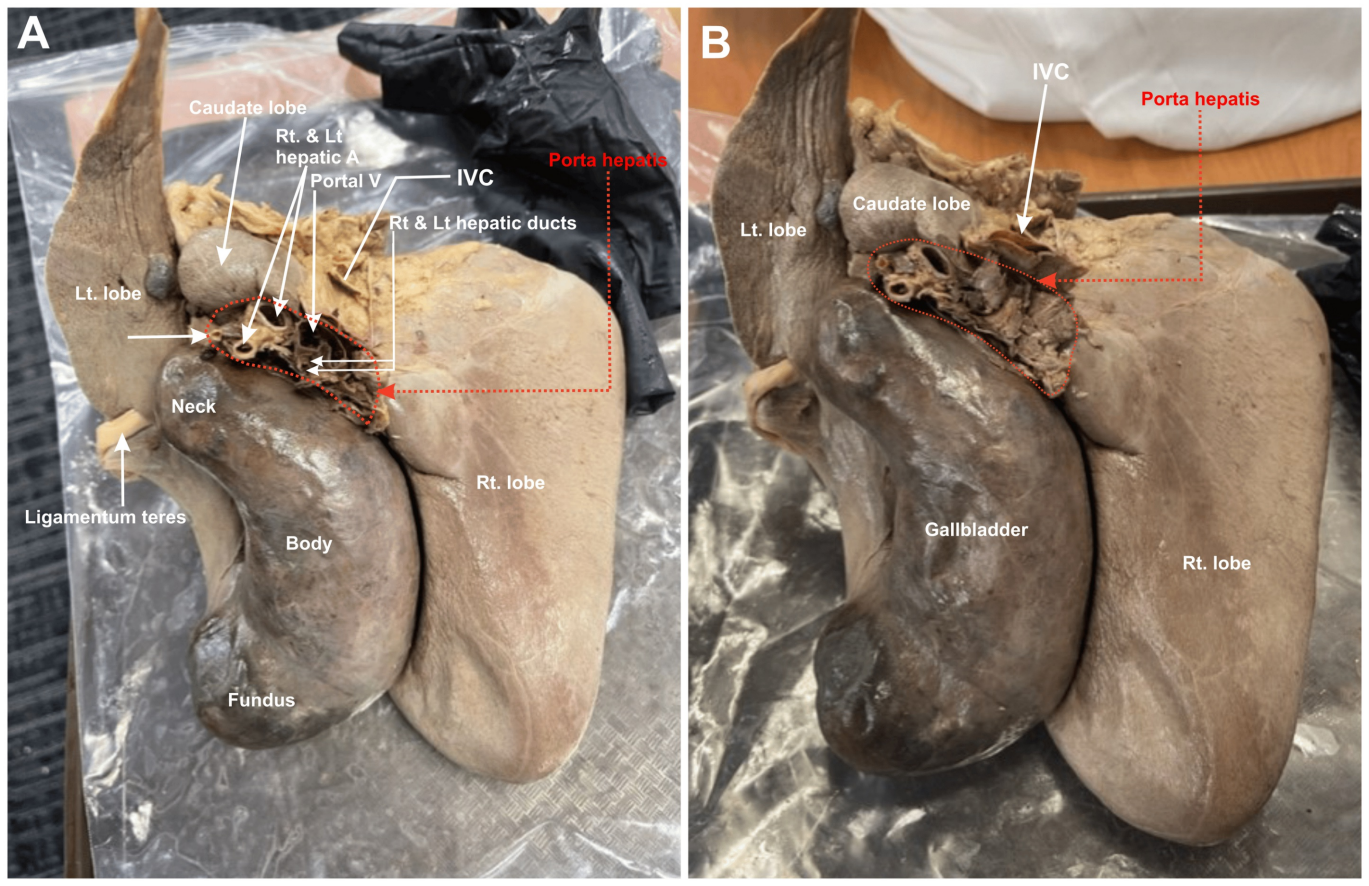
Inferior view of gross anatomy of fused liver and gallbladder. (A) The image highlights hepatic vasculature, lobes of the liver as well as neck, body, and fundus of the gallbladder. (B) The image further highlights hepatic vasculature including the inferior vena cava and porta hepatis. Rt: right; Lt: left; A: artery; V: vein; IVC: inferior vena cava

**Figure 3:**
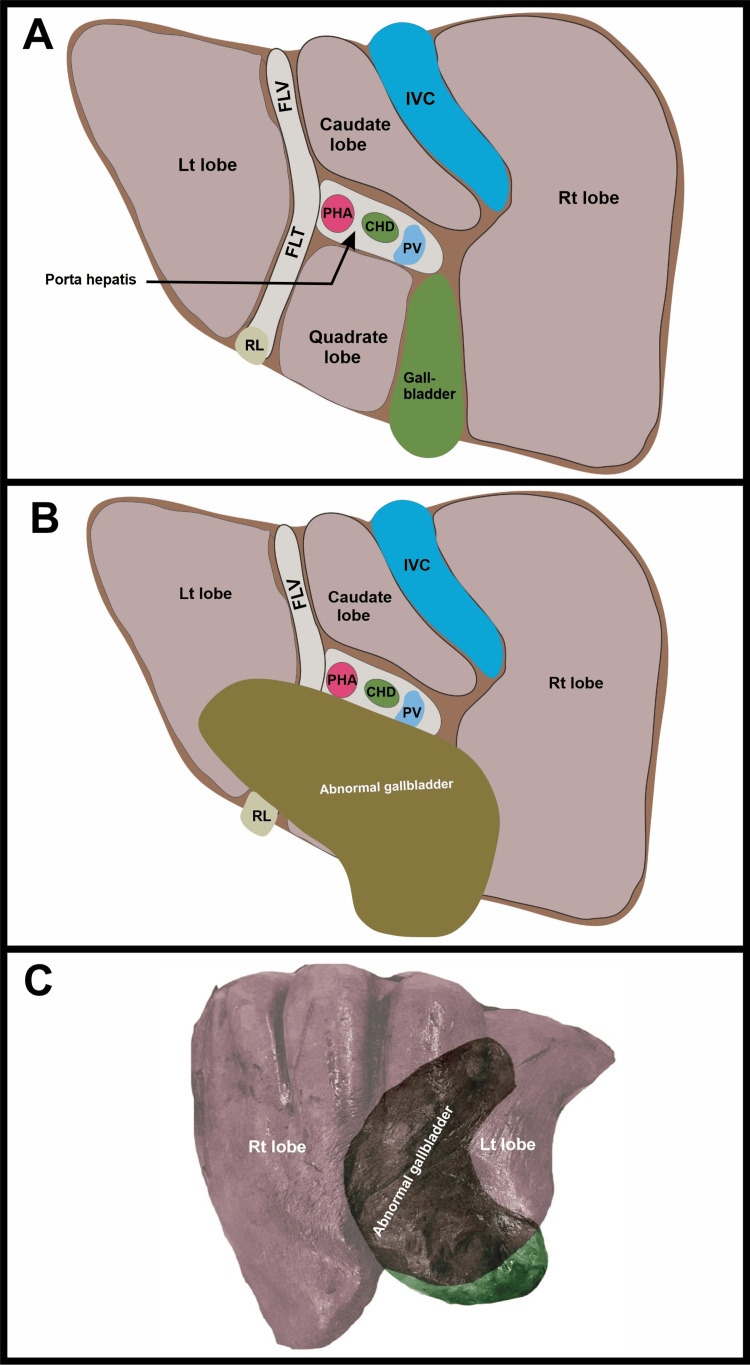
Illustration comparing case anatomy to a typical gallbladder and liver. (A) Illustration denoting the normal anatomy of the liver and gallbladder. (B) Anatomy of the gallbladder and liver in the current case report. (C) Superimposed outline of the gallbladder in Figure 1A. PHA: proper hepatic artery; CHD: common hepatic duct; PV: portal vein; IVC: inferior vena cava; RL: round ligament; FLV: fissure of the ligamentum venosum; FLT: fissure of ligamentum teres

The gallbladder and liver were firmly attached but able to be separated via blunt dissection (Figure 1). The total weight of the liver and gallbladder was 1,438 g, with the liver weighing 1,114 g and the gallbladder 324 g. The maximum vertical height of the liver was 18.2 cm and the maximum lateral measurement was 20.1 cm. The distal tip of the right lobe of the liver measured approximately 0.6 cm thick, and the depth of the four superior diaphragmatic furrows ranged from 0.5 to 1.2 cm. The gallbladder was 15 cm in length from the neck to the fundus, 5.5 cm wide, with a wall thickness of 0.1 cm. On the inferior aspect of the liver, the gallbladder could be seen obscuring the entire quadrate lobe (Figures 2, 3).

**Figure 4:**
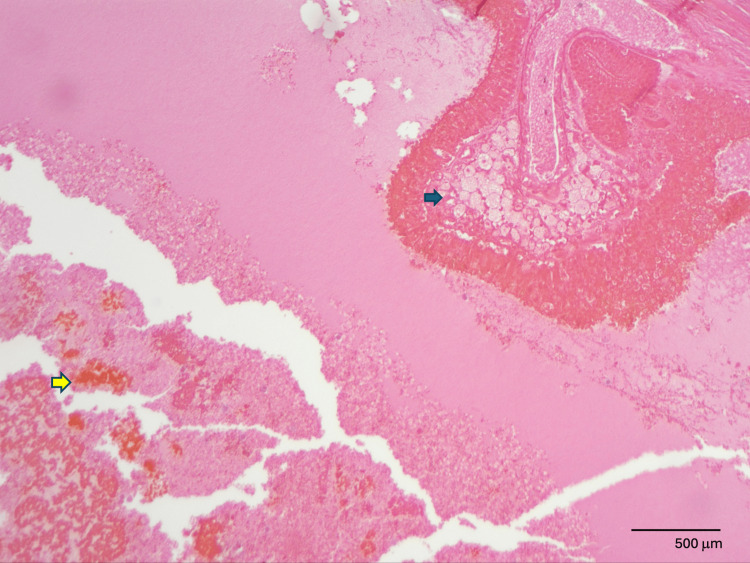
Histologic section of the gallbladder. Hematoxylin and eosin stained under 4× magnification. The figure demonstrates denuded epithelium, cholesterolosis (blue arrow), and luminal bile mixed with fibrin and degenerating red blood cells (yellow arrow).

**Figure 5:**
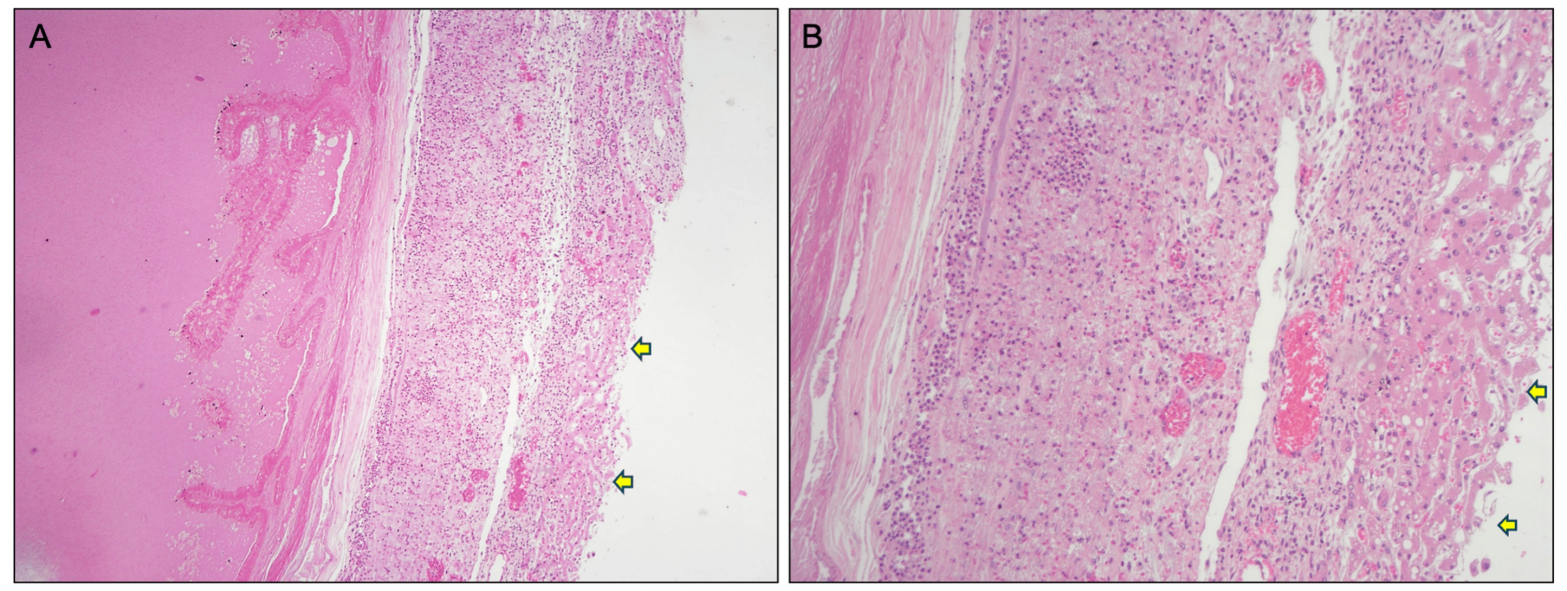
Low and high-power view of the area of fusion of the liver with the gallbladder. (A) Hematoxylin and eosin stained under 10× magnification. From left to right: gallbladder luminal contents, attenuated inner wall, acutely inflamed outer wall that is continuous with liver tissue (yellow arrows). (B) Hematoxylin and eosin stained under 20× magnification. From left to right: attenuated gallbladder muscularis, acutely inflamed outer wall that is continuous with liver tissue (yellow arrows).

Upon dissection, the gallbladder lumen was completely filled with a firm, dark red, beefy material. On histologic analysis by pathology, the material found in the lumen appeared to be composed of a mixture of bile, fibrin, and degenerating red blood cell products. The wall of the gallbladder demonstrated denuded epithelium with coagulative necrosis and cholesterolosis (Figure 4). Histological examination of the area of fusion found an acutely inflamed outer gallbladder wall that was adherent to the liver tissue via fibrous material (Figure 5). The cystic duct was difficult to identify, and when found, was exceptionally short. The diminutive cystic duct was only 2-3 mm long, with reactive epithelial cells lining the lumen.

## Discussion

The liver is a vital organ in the human body responsible for a wide range of functions that help support metabolism, digestion, immunity, detoxification, and vitamin storage among other functions [1]. The liver comprises roughly 2% of an adult’s body weight, but receives the most blood of any major organ in the body through the unique dual blood supply from the portal vein (approximately 75%) and hepatic artery (approximately 25%). The standard size of the liver is reported to be 10.5 cm in males and 7.0 cm in females, with greater or lesser than 2-3 cm in size being described as an abnormality. The liver typically weighs 1,400-1,500 g in an adult male and 1,200-1,400g in an adult female [9].

Diaphragmatic sulci, or grooves, are variations in the superior surface anatomy of the liver that have been reported in 15-40% of cases. More grooves are less common, with the presence of four grooves, as in the current case, reported in only 1% of cases [10-12]. Some researchers define these grooves as “cough furrows,” attributing their presence to coexisting respiratory disease that causes chronic cough. One study questioned whether furrows present in the sagittal plane are related to pulmonary causes, as they are not parallel to the anatomical course of the ribs [10]. The interaction between the diaphragmatic grooves and enlarged gallbladder in the current case report is difficult to determine, especially in the postmortem analysis of the specimen.

The gallbladder is an essential part of the digestive system; its main role is to store bile created by the liver. Bile is released in response to the release of the hormone cholecystokinin [3]. The liver and gallbladder arise as a single diverticulum from the summit of the developing duodenum. The summit then divides into two parts, the upper part called pars hepatica, which forms the liver, while the lower part is called pars cystica, which forms the gallbladder. The pars cystica is elongated, and the distal part of it widens to form the gallbladder, while the proximal part remains narrow to the cystic duct. Congenital dilatation of the entire gallbladder can result in loss of the cystic duct, and the gallbladder opens directly into the bile duct [13]. This congenital defect appears clearly in the present case. Normally, the gallbladder is a thin-walled, pear-shaped sack about 7 to 10 cm (2.7 to 3.9 inches) long and up to 5 cm (2 inches) across at its widest point [14]. Congenital malformations of the gallbladder have been described previously. Carbajo et al. in 1999 described 500 cholecystectomies, of which 1% had congenital anomalies [15]. The congenital anomalies reported were gallbladder and cystic duct agenesia (0.2%), left lobule misplacement with insertion of the cystic duct into the left hepatic duct (0.2%), and three gallbladder hypoplasias (congenital vesicle diverticula) (0.6%) [15]. Most congenital gallbladder diseases are asymptomatic and difficult to visualize on imaging [16]. In adults, there are several common gallbladder pathologies, including gallstones and gallbladder cancer [17]. Common reasons that a gallbladder may become enlarged is due to acute cholecystitis, which is due to blockage of the cystic duct [18]. The cadaveric specimen described in this case presented with a minuscule cystic duct measuring 2 mm in length. This finding potentially could have caused the cholecystitis in this patient as a shortened lumen can predispose patients to cystic duct obstructions. This highlights the importance of surgical consideration of such cases to avoid iatrogenic injury. This morbidity is associated with symptoms including right upper quadrant pain, fever, and nausea that may be associated with eating, and physical examination findings of right upper quadrant tenderness and fullness [3].

Intrahepatic gallbladder

Initially, it was believed that this discovered anomaly was an intrahepatic gallbladder. An intrahepatic gallbladder describes when a gallbladder lies within the liver parenchyma or has a subcapsular location on the anterior inferior right lobe of the liver [19]. Intrahepatic gallbladders are primarily caused by a rare congenital variant that results in the failure of the gallbladder to descend below the liver, but some instances have been reported secondary to chronic inflammation. One study has reported the incidence rate of an ectopic location of the gallbladder to be 0.1-0.7% [20]. While some patients can be asymptomatic, this unusual anatomy of the gallbladder can lead to hepatobiliary and gastrobiliary dysfunction resulting in hypertension, dyslipidemia, and jaundice. However, on histopathology microscopy, it was found that the gallbladder was not intrahepatic, but that the outer gallbladder wall was adherent to hepatic tissue via fibrous tissue marked with acute and chronic inflammation. The gallbladder displayed transmural acute inflammation, with the lumen exhibiting bile mixed with fibrin and degenerating red blood cells, and the surface epithelium markedly attenuated with areas showing coagulative necrosis and cholesterolosis. The liver showed evidence of cholestasis; however, there was a lack of fibrosis and no increased inflammation. This highlights the clinical importance of our findings of an adherent gallbladder and liver. Although the mechanisms of the anatomical variations are separate, there is potential for a gallbladder and liver fusion to cause similar pathologies. If radiologic images do not visualize the gallbladder then a fusion could be a rare differential to be considered.

Surgical implications

The volume of a normal gallbladder has an upper limit of 60 mL. Moderate enlargement of up to 200-300 mL is common in surgical practice due to various pathological conditions. When the gallbladder reaches this volume, the fundus of the gallbladder reaches the level of the anterior superior iliac spine. A giant gallbladder is a rare finding in which the structure has a weight exceeding 1.5 kg. In such cases, the gallbladder has lost its usual outlines and has acquired a balloon-shaped form. While the pathogenesis leading to this giant gallbladder remains unknown, many believe it is due to a valve-like mechanism associated with either stone(s), a tumor, or a “wandering gallbladder.” This leads to impaired drainage as well as unrestricted enlargement [8]. The clinical presentation of the giant gallbladder resembles a tumor or cyst of the abdominal cavity. The patient likely experienced symptoms of dull pain and fullness in the right upper quadrant of the abdomen [8]. The patient’s cystic duct was found to be extraordinarily small, which could have contributed to the signs of gallbladder inflammation found on histology. This finding could have potentially contributed to the amount of fibrin products exhibited by the gallbladder. The diminutive cystic duct could have led to this finding by obstructing bile outflow over time. This potential obstruction could also explain the findings of mixed acute and chronic inflammation. The clinical implications of these findings include the patient presenting with symptoms ranging from nothing to abdominal pain, fatty food intolerance, nausea, vomiting, indigestion, diarrhea, and jaundice. This patient may have had a normal physical examination, or they could have presented with a range of symptoms, including jaundice, localized tenderness over the gallbladder area, generalized abdominal tenderness, a palpable mass or fullness in the right upper quadrant of the abdomen, and potentially a positive Murphy’s sign. Recognizing this variant is important for surgeons performing laparoscopic cholecystectomy, liver transplant, tumor resection, and other hepatobiliary interventions to avoid possible complications.

## Conclusions

This case reports a rare presentation of an enlarged gallbladder adherent to the liver. Although believed to be a benign anomaly in this patient, the increased size of these two organs can potentially lead to pathologies such as biliary colic, mass effect, bloating, Budd-Chiari, and chronic and acute inflammation. Imaging modalities such as ultrasound, computed tomography, or magnetic resonance imaging can detect enlarged structures such as these, leading to opportunities to potentially combat or delay the negative implications of these enlarged organs. Therefore, clinicians should be aware of this variant and its clinical relevance. Organomegaly should be considered as a differential when treating patients with signs and symptoms of upper abdominal fullness or tenderness.
